# Meta smart glasses—large language models and the future for assistive glasses for individuals with vision impairments

**DOI:** 10.1038/s41433-023-02842-z

**Published:** 2023-12-04

**Authors:** Ethan Waisberg, Joshua Ong, Mouayad Masalkhi, Nasif Zaman, Prithul Sarker, Andrew G. Lee, Alireza Tavakkoli

**Affiliations:** 1 Department of Ophthalmology, University of Cambridge, Cambridge, UK; 2 Department of Ophthalmology and Visual Sciences, University of Michigan Kellogg Eye Center, Ann Arbor, MI USA; 3 University College Dublin School of Medicine, Belfield, Dublin Ireland; 4 Human-Machine Perception Laboratory, Department of Computer Science and Engineering, University of Nevada, Reno, Reno, NV USA; 5 Center for Space Medicine, Baylor College of Medicine, Houston, TX USA; 6 Department of Ophthalmology, Blanton Eye Institute, Houston Methodist Hospital, Houston, TX USA; 7 The Houston Methodist Research Institute, Houston Methodist Hospital, Houston, TX USA; 8 Departments of Ophthalmology, Neurology, and Neurosurgery, Weill Cornell Medicine, New York, NY USA; 9 Department of Ophthalmology, University of Texas Medical Branch, Galveston, TX USA; 10 University of Texas MD Anderson Cancer Center, Houston, TX USA; 11 Texas A&M College of Medicine, Bryan, TX USA; 12 Department of Ophthalmology, The University of Iowa Hospitals and Clinics, Iowa City, IA USA

**Keywords:** Health care, Business and industry

## Introduction

**Fig. 1:**
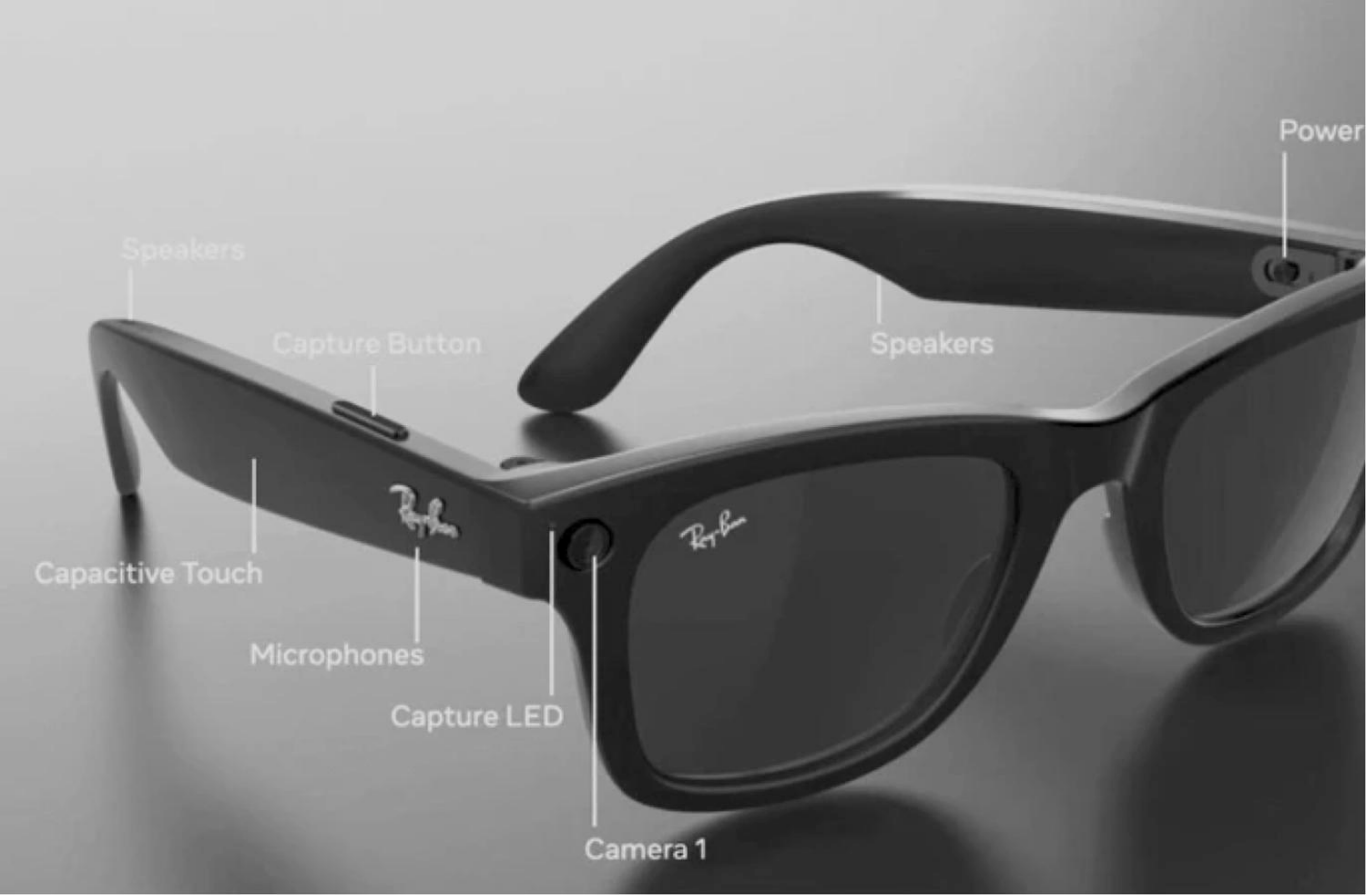
Technology components of Ray-Ban Meta smart glasses. Reprinted without changes from Laurent C, Iqbal, M.Z., Campbell, A.G. Adopting smart glasses responsibly: potential benefits, ethical, and privacy concerns with Ray-Ban stories. AI Ethics. under Creative Commons Attribution 4.0 International License http://creativecommons.org/licenses/by/4.0/.

In late September 2023, Meta unveiled its second generation of smart glasses in collaboration with Ray-Ban [1]. These smart glasses come with several improvements, including enhanced audio and cameras, over and a lighter design. The glasses are equipped with an ultra-wide 12 megapixel camera and immersive audio recording capabilities, allowing users to capture moments with a high level of detail and depth (Fig. 1) [1, 2]. These smart glasses are part of Meta’s efforts to develop AR and VR technologies. In addition, the glasses are equipped with AI-powered assistants like Meta AI [1].

Ray-Ban Meta smart glasses also represent a promising development in assistive technology for individuals with visual impairments and have the potential to significantly enhance their quality of life. The field of assistive technology has been advancing rapidly in recent years, particularly due to significant advances in artificial intelligence [3] and augmented reality [4]. Envision is currently one of the leading smart glasses developers, and their technology allows visual information to be articulated into speech for individuals with vision impairments. A recent update included GPT integration, allowing users to ask the glasses specific questions, like to summarize text, or only reading vegan items from a menu. GPT-4 [5]. Future updates will further increase the usefulness of this integration [6].

The Envision smart glasses are built on the Google Glass Enterprise Edition 2 (now discontinued), and the high price of the Google smart glasses likely posed as a barrier of the adoption to this helpful technology in vision impaired individuals. Lowering the cost of assistive technologies is essential, as previous research in the UK found a staggeringly low employment rate of 26% for blind and partially sighted working age individuals [7].

**Fig. 2:**
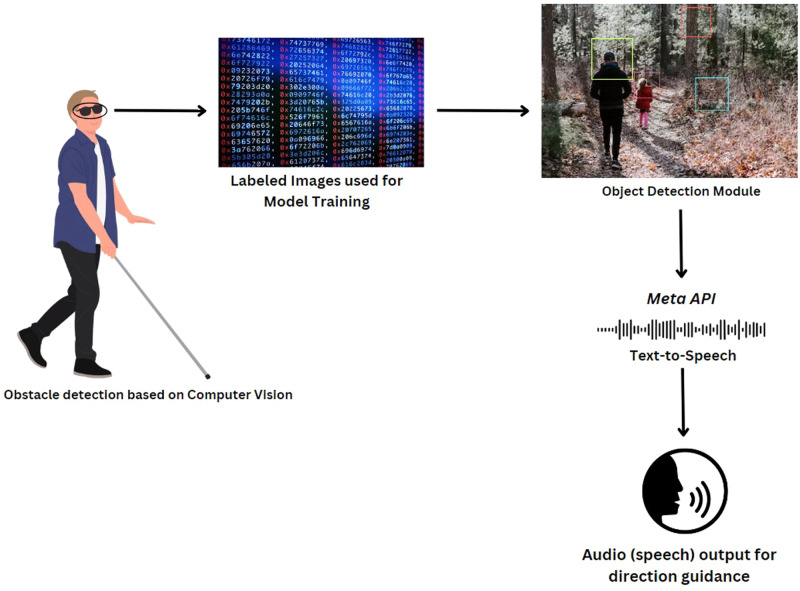
Diagram of how smart glasses can provide auditory direction guidance for individuals with vision impairments.

By Meta attempting to make smart glasses a mainstream technology, the cost of smart glasses will continue to decrease in the coming years. The incorporated advanced camera technology can provide real-time image processing, while the built in AI can recognize objects and convert this visual information into speech [1]. An update planned within the next year is expected to allow users to ask Meta AI questions about what they are looking at. Users can potentially interact with these assistants to receive auditory information about their environment, read text aloud, recognize faces, or get directions, which can be invaluable for individuals with visual impairments (Fig. 2). Future incorporation of GPS navigation accompanied with audio cues facilitates self-navigating for individuals with visual impairments in new environments. Previous research in the U.K. showed that nearly 40% of blind and partially sighted individuals are not currently able to complete all of the journey that they need or wish to make [7]. Better accessibility through the usage of smart glasses can lead to greater independence for individuals with vision impairments.

Meta hopes to incorporate augmented reality in future versions of smart glasses, and describes the current stage as a stepping stone to true augmented reality. Users with vision impairments would benefit highly from true augmented reality glasses, with potential features like magnification, contrast enhancement, and color correction, enhancing their ability to see and navigate their surroundings more effectively. Meta’s future augmented reality work will be compared to the Apple Vision Pro, which is also looking to make mixed reality devices mainstream [8, 9]. Further research will also be required to minimize the variability between various different VR/AR devices prior to clinical use [10]. We look forward to continued advances in augmented reality with AI integration, and believe this technology can revolutionize how individuals with vision impairments interact with the world.
